# AIxSuture: vision-based assessment of open suturing skills

**DOI:** 10.1007/s11548-024-03093-3

**Published:** 2024-03-25

**Authors:** Hanna Hoffmann, Isabel Funke, Philipp Peters, Danush Kumar Venkatesh, Jan Egger, Dominik Rivoir, Rainer Röhrig, Frank Hölzle, Sebastian Bodenstedt, Marie-Christin Willemer, Stefanie Speidel, Behrus Puladi

**Affiliations:** 1 Department of Translational Surgical Oncology, NCT/UCC Dresden, Dresden, Germany; 2 The Centre for Tactile Internet (CeTI), TUD Dresden University of Technology, Dresden, Germany; 3 Department of Oral and Maxillofacial Surgery, University Hospital RWTH Aachen, Aachen, Germany; 4 Institute for AI in Medicine, University Hospital Essen (AöR), Essen, Germany; 5 School of Embedded Composite Artificial Intelligence (SECAI), TUD Dresden University of Technology, Dresden, Germany; 6 Institute of Medical Informatics, University Hospital RWTH Aachen, Aachen, Germany; 7 MITZ, University Hospital Carl Gustav Carus, TUD Dresden University of Technology, Dresden, Germany; 8 Faculty of Medicine, University Hospital Carl Gustav Carus, Dresden, Germany; 9 German Cancer Research Center (DKFZ), Heidelberg, Germany; 10 BMBF Research Hub 6 G-Life, TUD Dresden University of Technology, Dresden, Germany

**Keywords:** Surgical skill training, Suturing, Open surgery

## Abstract

**Purpose:**

Efficient and precise surgical skills are essential in ensuring positive patient outcomes. By continuously providing real-time, data driven, and objective evaluation of surgical performance, automated skill assessment has the potential to greatly improve surgical skill training. Whereas machine learning-based surgical skill assessment is gaining traction for minimally invasive techniques, this cannot be said for open surgery skills. Open surgery generally has more degrees of freedom when compared to minimally invasive surgery, making it more difficult to interpret. In this paper, we present novel approaches for skill assessment for open surgery skills.

**Methods:**

We analyzed a novel video dataset for open suturing training. We provide a detailed analysis of the dataset and define evaluation guidelines, using state of the art deep learning models. Furthermore, we present novel benchmarking results for surgical skill assessment in open suturing. The models are trained to classify a video into three skill levels based on the global rating score. To obtain initial results for video-based surgical skill classification, we benchmarked a temporal segment network with both an I3D and a Video Swin backbone on this dataset.

**Results:**

The dataset is composed of 314 videos of approximately five minutes each. Model benchmarking results are an accuracy and F1 score of up to 75 and 72%, respectively. This is similar to the performance achieved by the individual raters, regarding inter-rater agreement and rater variability. We present the first end-to-end trained approach for skill assessment for open surgery training.

**Conclusion:**

We provide a thorough analysis of a new dataset as well as novel benchmarking results for surgical skill assessment. This opens the doors to new advances in skill assessment by enabling video-based skill assessment for classic surgical techniques with the potential to improve the surgical outcome of patients.

**Supplementary Information:**

The online version contains supplementary material available at 10.1007/s11548-024-03093-3.

## Introduction

It is well known that improving surgical skill greatly improves patient outcome [1]. Common practice in surgical training is for residents or medical students to assist attending surgeons in the operating room (OR). Attending surgeons or mentors then give feedback based on their subjective observations of the resident in the OR. The issue within this is that involvement in the surgical procedure is coupled to a resident’s surgical training. Therefore, the feedback given in this setting is greatly subjective since it depends entirely on the mentoring attending surgeon. This leads to unstructured, inconsistent, and subjective training, consequently, resulting in ineffective learning for trainees. For this purpose, standardized evaluation assessments, procedural checklists, and rating scales have been established [2–4].

To further standardize surgical training it is essential to automate training and feedback mechanisms [5]. Typically, surgical mentors have little time and are costly. To bridge this gap, machine learning models are being developed and adapted to the context of surgical skill assessment. Current popular methods used for surgical skill assessment can be divided into two categories: motion based and video based [6]. Motion-based approaches involve preprocessing the data by extracting or measuring features such as instrument motion [7, 8], force and torque measurements [9], robot kinematics [9], or even eye-tracker data [10].

Motion-based approaches typically require additional preprocessing, sensors, or data collection. In contrast, video-based methods do not require additional hardware or steps which also facilitates the translation into clinical practice. Funke et al. [11] combined a temporal segment network (TSN) [12] and a 3D convolutional neural network (CNN) to classify surgeons using robotic instruments into three skill levels using only video data. Anastasiou et al. [13] classified the videos using a ResNet and a temporal component network to extract features which are given to a transformer utilizing contrastive learning. Kiyasseh et al. [14] also utilized a vision transformer to classify a surgeon into low and high skill categories.

In general transformers have recently captivated the community. However, few have applied them to surgical skill assessment. Kiyasseh et al. [14] trained a vision transformer for their task. Vision transformers are specialized for image-based applications. Beyond this, a video shifted window (Swin) transformer was developed by Liu et al. [15]. They extend the original Swin transformer to apply the local shifting window in the spatial domain to the spatiotemporal domain. It is therefore specialized to learn temporally encoded information prevalent in skill assessment tasks.

All of these approaches involve minimally invasive (MI) procedures. This is due to the fact that MI surgery requires some form of camera from which video data can be easily collected for further analysis. This is not the case for open surgery. Additionally training for open surgery is still widely unstandardized [16].

However, receiving objective feedback on training is arguably significantly more important for open surgery given that all practicing surgeons must obtain and hone these skills. Surgical skill assessment datasets for open surgery techniques are rare due to the prospect that videos are not as easily obtained when compared to minimally invasive techniques [3, 17, 18].

Approaches so far for open surgical skill assessment have been largely simulation based [3, 17]. Similarly to Fard [7], Goldbraikh et al. extracted features—duration, path length, and number of movements—to assess a surgeon’s skill. They use a YOLO-based model to track instruments and surgeons’ hands. Then, they calculate the metrics based on the tracked data. Alternatively, Kil et al. [17] collected external data from which to determine a surgeon’s skill. They developed a simulator which collects synchronized force, motion, video, and touch data.

Further works on open surgery skill assessment are quite sparse. Therefore to the best of our knowledge, a model classifying the surgical skill directly from a video using an open surgery dataset without the requirement of hand-crafted feature extraction has not yet been published. Avoiding hand-crafted feature extraction and additional data collection steps saves time and resources while also capturing nuances in the videos which may go unnoticed otherwise. Furthermore, a video dataset with labeled skill ratings has not yet been benchmarked for open surgical techniques. In this paper, we present a large dataset of open suturing recordings that we released in a recent publication [19]. We provide an in-depth analysis of the data as well as benchmarks for automatic skill assessment, including the first end-to-end trained approach for skill assessment for open surgery training.

## Dataset

### Description

**Fig. 1:**
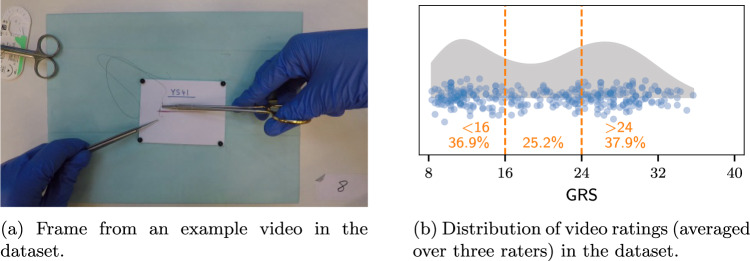
The AIxSuture dataset contains 314 expert-rated videos of open suturing training, recorded at the University Hospital in Aachen, Germany. We distinguish proficient (GRS $\ge$ 24), novice (GRS < 16), and intermediate skill levels (16 $\le$ GRS < 24)

The AIxSuture dataset is the result of a study we recently published [19, 20]. It is comprised of 314 videos of approximately 5 min each taken at 30 fps resulting in about 100 GB of video material. The videos are taken in a standardized setting with a GoPro Hero 5 from a bird’s-eye-view and have no camera motion. The videos are recorded of students performing open surgery suturing in a simulated setting. An example image of a video from the dataset can be seen in Fig. 1a.

The data was collected to analyze the effectiveness of virtual reality head-mounted, display-guided training on medical and dental students. This previous study was performed to compare the virtual reality supported training to an e-learning and tutor-based approach. Therefore, a pre- and post-training video was taken from each student and rated with the according scores. Each student and video has its own identifier. For each video, the corresponding student IDs are recorded in an Excel spreadsheet together with the skill scores rated by three independent raters. Skill scores are recorded using the Objective Structured Assessment of Technical Skills (OSATS) [21] scale with eight skill categories. The sum of all categories forms a global rating score (GRS), ranging from scores of 8 to 40. An initial analysis of the inter-rater reliability of three raters yielded an average pairwise Pearson correlation coefficient of $> 0.8$ [20].

In this paper, to provide an initial benchmark for the data we focused on the GRS scores. For each video, the individual rater scores were averaged and subsequently categorized into three classes—novice, intermediate, and proficient. We distinguish proficient (GRS $\ge$ 24), novice (GRS < 16), and intermediate skill levels (16 $\le$ GRS < 24). In practice, an OSATS score greater than 24 is considered proficient in skill demonstration which we use as one of our class delimiters. We chose the midpoint between 8 and 24 for the class delimiter between the novice and intermediate class.

Consequently, this class division does result in a slightly imbalanced dataset regarding the intermediate class. This is also due to the dataset collection process. Since the videos were recorded before and after one hour of training for each participant the skill improvement is clearly apparent, directly resulting in the lower density of points between 16 and 24 seen in Fig. 1b.

We propose to split the dataset into training, validation, and test set, using 70% of the data for training, 15% for validation, and 15% for testing. Importantly, pre- and post-training videos of the same student, identified by the unique student identifier, are kept within one subset of the data. This way the model is ensured to generalize across different students’ suturing styles and techniques. Each video is included only once, and the split sets are disjoint. Within these splits, the beginner and proficient classes are each comprised of 30–40% of the data, and the intermediate class is approximately 20–30%. An exact notation of the videos included in each split set is provided in the Supplementary Material.

### Inter-rater agreement analysis

We analyzed the dataset in order to contextualize model behavior and promote best practices for model design, evaluation, and hyperparameter tuning. Inter-rater agreement is an essential aspect for adequate skill assessment. Not only does it promote objectivity in the ratings and, consequently, in the model’s objectivity, but also in model performance.

**Fig. 2:**
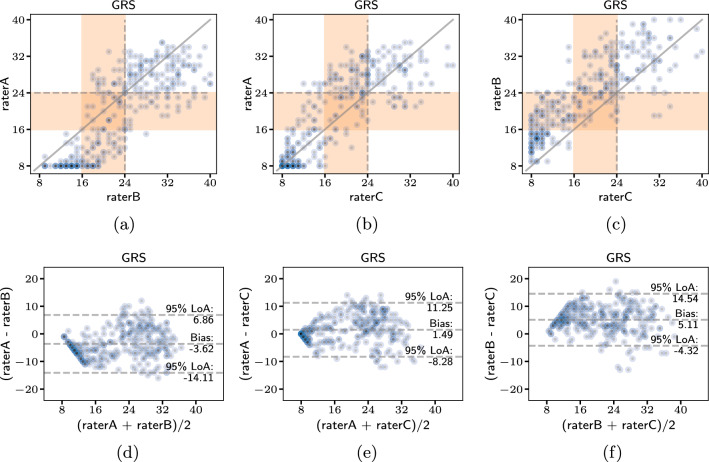
Plots comparing the individual rater scores to each other. The top row (a–c) depicts the direct GRS scores given by each rater pair. The bounds of the assigned class are denoted by the orange region. The bottom row (d–f) presents Bland–Altman plots with limits of agreement

While the dataset has an excellent inter-rater agreement with an average pairwise Pearson correlation coefficient of 0.8, the raters are nonetheless individually slightly biased. As can be inferred from Fig. 2a and d, rater A typically rated surgeons significantly lower than rater B. This is especially apparent in the pre-training scores. Rater A and rater C generally had more agreement which can be seen in Fig. 2b and e, although as skill level increased, rater A tended to give higher scores than rater C. Similarly, rater B rates videos significantly better than rater C as can be seen from Fig. 2c and f.

What all images in Fig. 2 reflect is that raters have a better consensus on lower skill levels than higher ones. This is especially well distinguishable in the Bland–Altman plots. The differences between scores for all raters are less for average scores lower than 16. Additionally, as visualized by the distribution of scores in Fig. 1b, it is easier for raters to distinguish higher and lower skill levels than intermediate skill level. The distribution also reflects this tendency since more scores in lower and higher regions are given by the raters than in middle/intermediate section. However, it must be kept in mind that the nature of the study from which the dataset originated influenced theses scores. For each participant, a video was taken and rated before and after an hour of training. Therefore, this is also encased in the score distribution.

## Benchmarking

### Methods

We evaluate two different methods for skill assessment on the AIxSuture data [19]. The first is a state of the art model developed by Funke et al. [11] using an I3D model in combination with a temporal segment network (TSN) [12]. The other is an adapted version of the network in which the I3D network backbone has been replaced by a Video Swin transformer. We will further refer to the Video Swin transformer simply as Video Swin. The I3D and Video Swin backbones are pretrained with the Kinetics400 [22] dataset.

**Backbones** The backbones chosen were the I3D and Video Swin models. The I3D model is a CNN-based network that considers the temporal information encoded in stacks of consecutive video frames. Essentially, this model is a variant of the Inception model in which the convolutional filters, pooling operations, and feature maps have a third dimension [11]. Similarly, the Video Swin model is an extension of the vision transformer (ViT). ViTs use self-attention to capture long-range pixel dependencies. The Video Swin further applies a sliding window in the visual as well as the temporal plane to capture dependencies between image and frame sequence patches [15].

**Preprocessing** From the dataset videos, frames were extracted at 5 fps. The extracted frames are resized to 270x480 pixels for better data handling and loading. The models were trained on the three classes determined by the GRS score.

**Fig. 3:**
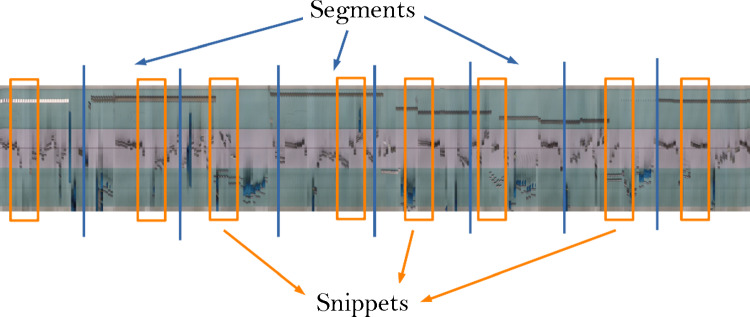
To train the TSN, a sample video is first divided evenly into segments. From each segment, one snippet is extracted at a random position. Each snippet has the same frame length

**Fig. 4:**
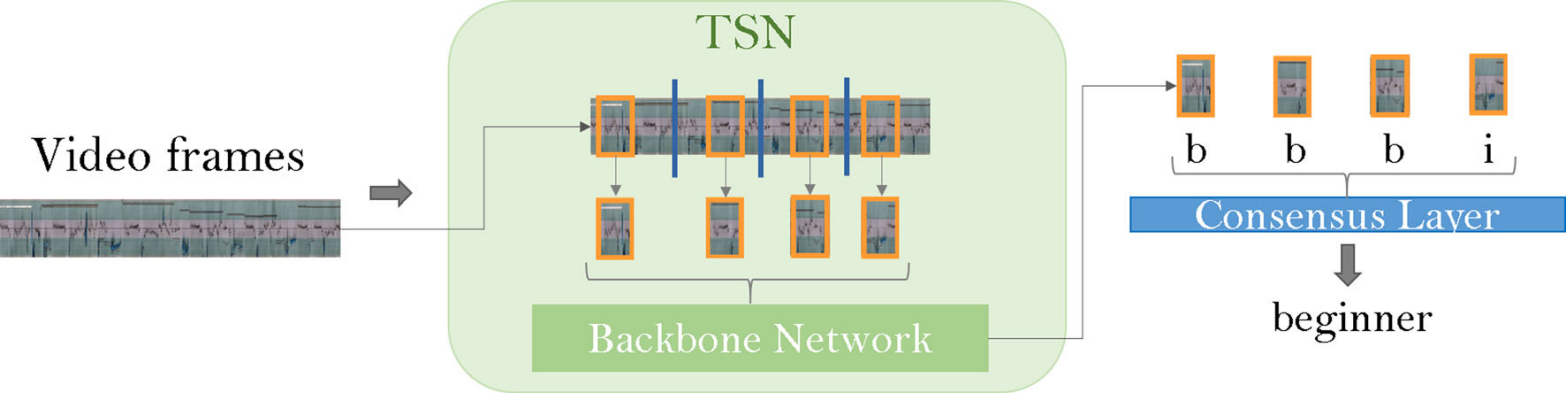
Schematic showing the network flow overview. A video is separated into frames and given to the TSN. This divides the video further into snippets (highlighted in orange), and passes these to the backbone—either an I3D or Video Swin model. Each snippet is individually evaluated (e.g., b—beginner, i—intermediate) and then merged into one rating for the entire video

**Model Workflow** The methods for skill assessment follow the TSN framework: during training a video is divided evenly into a given number of segments. From each segment, a snippet of frames is extracted from a random position within the segment. Each snippet has a predefined number of frames denoted by the snippet length. This process has been exemplified in Fig. 3. Snippet lengths and number of segments are chosen accordingly that approximately half of the video is seen by the model during training. All snippets from one video are combined in a batch and evaluated independently using the backbone model. This is schematically shown in Fig. 4. The data is, therefore, input into the system as a (B $\times$ S) $\times$ C $\times$ T $\times$ H $\times$ W tensor, in which the height H and width W of a frame are equal, B represents the chosen batch size, $C=3$ for the number of RGB channels, T is the snippet length, and S denotes the number of segments. A consensus layer averages the results from all snippets of a video, and the cross entropy loss over the classification error is calculated and back propagated through the network. In this way, the network learns to correctly classify each video by correctly classifying the snippets. During evaluation, the snippet length and number of segments are chosen that the full video is seen by the model.

**Experimental details** For the I3D network, the last three layers are unfrozen during training. Due to hardware restrictions, only the final stages of the Video Swin models could be trained, while the rest remained frozen. This included two transformer blocks.

We benchmarked various Video Swin model sizes, including Video Swin Tiny, Small, and Big. Each experiment was performed three times. In each experimental run, we trained a model for 400 epochs and selected the model weights that had achieved the best F1 score in the validation set. We also tested different hyperparemeter configurations of varying snippet lengths and number of segments, dropout rates, number of unfrozen model layers, and learning rates.

### Results

**Automatic assessment** Overall performance of the models was good, achieving F1 scores of up to 72% and accuracies of 75%. For all tested backbones, model performance varied by class as can be seen in Table 1. Novice and proficient classes were easier for the models to classify than the intermediate class.

**Table 1:** Classwise F1 score of models on the unseen test set

Model	Novice	Intermediate	Proficient	Macro Average
I3D	**0**.**8571**	0.4030	0.7514	0.6917
Swin Tiny	0.8373	**0**.**5378**	**0**.**7723**	**0**.**7157**
Swin Small	0.7907	0.3713	0.7317	0.6313
Swin Big	0.8357	0.3724	0.6986	0.6356

Swin refers to the Video Swin transformer. Best results per class are bolded

In Table 2, the comparisons of the different models as well as their combinations of snippets and segments are shown. The Video Swin Tiny and I3D models are stronger in performance in comparison with the Video Swin Big and Small. We hypothesize that this is due to possible overfitting and inability to generalize to the data by the Video Swin Big and Small models. This could be caused by the size of the models and the ratio of trainable to total model parameters. The Tiny model has a ratio of 51.34% while that of the Small and Big models are below 30% due to hardware limitations.

Varying the snippet length and the amount of segments had subtle effects on model performance. Only a small variability within the chosen number of segments was possible due to GPU memory constrictions. Surprisingly, increasing the snippet length, and thereby increasing the amount of information the model sees at one time, does not result in increased model performance across the board. The I3D and Video Swin Tiny models showed better performance with shorter snippet lengths. Decreasing snippet length and increasing the amounts of segments places focus on the details. This suggests that the details of the actions and movements gained from shorter video snippet lengths aid the skill assessment interpretation of these models.

**Table 2:** Average results of varying snippet lengths and segments on the test set

Model	Snippets	Segments	Validation F1	Test F1	Test Accuracy
I3D	64	10	0.789 ± 0.021	0.647 ± 0.028	0.674 ± 0
**I3D**	64	12	**0.804 ± 0.003**	**0.692 ± 0.029**	**0.746 ± 0.063**
I3D	75	10	0.787 ± 0.020	0.629 ± 0.023	0.645 ± 0.013
Swin Tiny	64	10	0.817 ± 0.004	0.634 ± 0.081	0.681 ± 0.070
**Swin Tiny**	64	12	**0.808 ± 0.010**	**0.716 ± 0.043**	**0.732 ± 0.033**
Swin Tiny	75	10	0.804 ± 0.037	0.661 ± 0.094	0.688 ± 0.100
Swin Small	64	10	0.749 ± 0.014	0.574 ± 0.024	0.623 ± 0.033
Swin Small	64	12	0.739 ± 0.173	0.604 ± 0.014	0.631 ± 0.0
**Swin Small**	75	10	**0.751 ± 0.033**	**0.631 ± 0.059**	**0.659 ± 0.066**
Swin Big	64	10	0.752 ± 0.017	0.608 ± 0.083	0.646 ± 0.087
**Swin Big**	64	12	**0.773 ± 0.065**	**0.636 ± 0.032**	**0.652 ± 0.038**
Swin Big	75	10	0.776 ± 0.006	0.607 ± 0.058	0.623 ± 0.045

Metrics are macro-averaged over all classes. Swin refers to the Video Swin transformer

Bold indicates best performance per model architecture

In conclusion, we recommend using the Video Swin Tiny model as a backbone. It is able to outperform the Small and Big models while requiring significantly less GPU memory. While the I3D performs similarly in accuracy, its F1 score is lower than that of the Video Swin Tiny model. The Video Swin Tiny model retains similar F1 and accuracy scores, showing that it is able to overlook the class imbalance of the dataset. However, in case of memory restraints, the I3D is an elegant choice. It requires significantly less memory than even the Video Swin Tiny model. Though with this model choice, a slight performance drop should be expected by the user.

**Comparison to human raters** We analyzed the performance of each rater for each class. This was done with the purpose of baseline comparability of our models. To assess individual rater performance with regard to the ground truth skill classes, we discretized the rater’s GRS scores into the three classes (novice, intermediate, proficient) using the same delimiters as described before. Then, we compared the rater’s classifications with the true classes by computing class-wise F1, macro-averaged F1, and accuracy on the test videos. The results of which are in Table 3. Overall, scoring by rater A was the closest to the ground truth classification for a given video.

**Table 3:** Rater F1 scores on the test set compared to the F1 scores of the Video Swin Tiny model

	Novice	Intermediate	Proficient	F1	Accuracy
Rater A	0.9545	0.5785	0.8513	0.7978	0.8399
Rater B	0.7007	0.5301	0.8854	0.7054	0.7391
Rater C	0.8804	0.4879	0.7519	0.7067	0.7391
Rater Mean	0.8452	0.5321	0.8295	0.7356	0.7727
Swin Tiny	0.8373	0.5378	0.7723	0.7157	0.7319

F1 and accuracy are macro-averaged over all classes in the last two columns

**Fig. 5:**
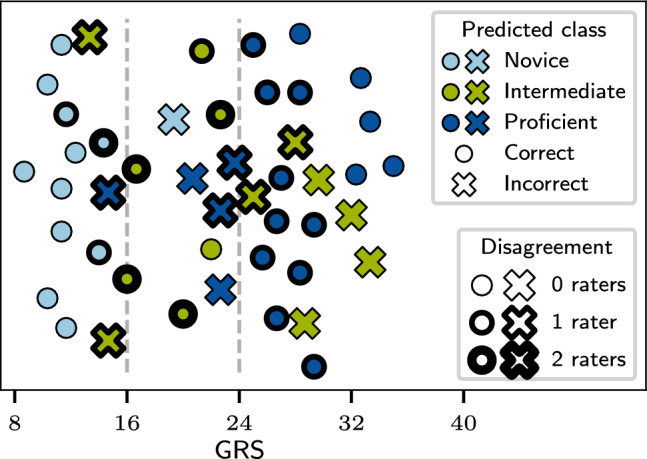
Predictions of the Video Swin Tiny model on the test set. Each data point refers to one test video. Model predictions of the three experimental runs are fused using majority voting. The x-coordinate of a marker corresponds to the ground truth GRS of the video, while the color presents the skill level that was assigned by the model. Incorrect classifications are marked by crosses. The edge width of a point represents whether one or two raters had assigned the video to a different skill level, thus deviating from the ground truth

Similarly to model performance, the rater metrics classifying the intermediate class were significantly lower than for novice and proficient classes. We hypothesized whether the lacking model performance for the intermediate class was due to border cases for which the average GRS score falls on or close to one of the two class borders—16 and 24. We investigated this with the Video Swin Tiny model. Results of which are shown in Fig. 5. From the figure, it can be seen that misclassifications are typically involved with the border cases, especially when differentiating between the intermediate and proficient classes. However while investigating this, we also discovered that model performance was also tied to the rater disagreement. The Video Swin Tiny model classified fourteen videos incorrectly. Of those misclassifications, seven videos had a disagreement of at least one rater.

## Discussion

The dataset is an excellent start in enabling automatic skill assessment for open surgery techniques. Our video-based method makes a clear distinction from other open surgery skill assessment methods by directly learning feature representations from video data. We thereby omit intermediate hand-crafted feature extraction, saving time and resources while also simplifying the process. This in turn eases the way for translation of skill assessment methods into practice.

Furthermore, other studies only divide their skill predictions into binary classes of high/good and low/bad skill. This is neither precise nor informative feedback for the person being evaluated. With the prospect of moving toward a full OSATS rating, we chose to divide our skill classifications into three categories. This would greatly impact the medical community by providing structured training and feedback. Classifying skill into three instead of two classes makes the classification task harder, explaining the somewhat lower metrics scores in comparison with other skill evaluation algorithms. The finer-grained the rating, the more variability exists. This was also apparent in the model results and also in the rater assessments. This challenge is demonstrated in Table 3 and Fig. 5. The intermediate class was the most difficult to interpret across the board. This is in part due to the distribution of the data and the class divisions, but also on the subtle but present rater disagreement. However, as can also be inferred from Table 3, the model performance is comparable to the raters.

We specifically chose not to implement any learning rate schedulers and further optimizations to provide a baseline benchmark. Future research could consider implementing these training strategies to optimize model performance. Further exploration should also be considered regarding the influence of snippet length and segments.

A further limitation of the dataset is that it is composed mainly of good and very bad performances due to the pre- and post-training setup. In order to achieve a more homogenous distribution, the dataset requires more recordings of intermediate and expert (GRS $>32$) performances.

## Conclusion

In this paper, we present benchmarks for a new dataset for open suturing training. The dataset consists of 314 suturing videos of various surgeons with differing skill levels. Each video is annotated accordingly with respect to the OSATS rating scale.

We benchmarked the dataset with two models using a TSN base architecture. The backbones were replaced by an I3D network and a Video Swin transformer, both of which have not yet been applied for open surgery skill analysis. Furthermore, we are the first to predict surgical skill for open surgery based only upon video data. No preprocessing—other than resizing—or other data extractions were performed on the videos prior to submitting them to the models.

Our work sets the baseline for future progress in open surgical skill assessment predictions from videos upon which future work can build and improve. By also including a skills assessment beyond a binary classification, we provide the essential start to developing finer-grained skill assessment, and working our way toward a full GRS or even full OSATS predictions.

### Supplementary Information

Below is the link to the electronic supplementary material.

**Supplementary information**This article has an accompanying supplementary file. (pdf 81KB)

## Data Availability

https://zenodo.org/record/7940583.

## Code availability

https://gitlab.com/nct_tso_public/aixsuture.
