# Supplementary material for: AIxSuture: vision-based assessment of open suturing skills
Source: Int J Comput Assist Radiol Surg. 2024 Mar 25;19(6):1045–52. doi: 10.1007/s11548-024-03093-3 (PMC11178625; doi:10.1007/s11548-024-03093-3)
Supplement: Supplementary file 1 — Supplementary informationThis article has an accompanying supplementary file. (pdf 81KB) [file 11548_2024_3093_MOESM1_ESM.pdf]

AIxSuture: Vision-Based Assessment of Open Suturing Skills

Hanna Hoffmann[1,2,8*], Isabel Funke[1,2], Philipp Peters[3], Danush Kumar Venkatesh[1,5,8], Jan Egger[4], Dominik Rivoir[1,2], Rainer Röhrig[6], Frank Hölzle[3], Sebastian Bodenstedt[1,2], Marie-Christin Willemer[7,8], Stefanie Speidel[1,2,8†], Behrus Puladi[3,6†]

[1*]Translational Surgical Oncology, NCT/UCC Dresden, TU Dresden.
[2]The Center for Tactile Internet (CeTI), TU Dresden.
[3]Oral and Maxillofacial Surgery, University Hospital RWTH Aachen.
[4]Institute for AI in Medicine, University Hospital Essen (AöR).
[5]SECAI, TU Dresden.
[6]Institute of Medical Informatics, University Hospital RWTH Aachen.
[7]MITZ, University Hospital Carl Gustav Carus, TU Dresden.
[8]Faculty of Medicine, University Hospital, TU Dresden.

*Corresponding author(s). E-mail(s): hanna.hoffmann@nct-dresden.de;
†These authors jointly supervised this work.

1 Hardware and Software

Model training and evaluation took place on an NVIDIA RTX A5000 and an NVIDIA A100 GPU with 24 and 80 GB RAM, respectively. While the I3D network could run on the A5000, the SWIN transformer models were too large to process with the limited space of the A5000 GPU and were therefore processed with the A100. All code is written and tested under python 3.8 using CUDA/torch version 11.8.

2 Splits

Train, test, and validation splits were created using the torch random generator with a seed of 42.

Train split: ['D94Z', 'Z49D', 'R65M', 'M56R', 'D44W', 'W44D', 'Z13N', 'N31Z', 'R37W', 'W73R', 'R48T', 'T84R', 'U79I', 'I97U', 'C28D', 'D82C', 'A59Z', 'Z95A',

'K71Q', 'Q17K', 'T25P', 'P52T', 'D17H', 'H71D', 'C43T', 'T34C', 'V11M', 'M11V', 'O16G', 'G61O', 'T45Y', 'Y54T', 'X49O', 'O94X', 'Y65C', 'C56Y', 'Q38Q', 'Q83Q', 'C42I', 'I24C', 'Z62S', 'S26Z', 'B53D', 'D35B', 'K53Y', 'Y35K', 'Q58C', 'C85Q', 'V63F', 'F36V', 'X94Z', 'Z49X', 'T98Q', 'Q89T', 'P54M', 'M45P', 'P55V', 'V55P', 'S93I', 'I39S', 'G22R', 'R22G', 'C36X', 'X63C', 'L13Q', 'Q31L', 'T54X', 'X45T', 'A99Y', 'Y99A', 'D12Y', 'Y21D', 'X99A', 'A99X', 'S33L', 'L33S', 'Q43O', 'O34Q', 'N49S', 'S94N', 'R55K', 'K55R', 'W38P', 'P83W', 'N75S', 'S57N', 'R77V', 'V77R', 'G66T', 'T66G', 'H27O', 'O72H', 'P77F', 'F77P', 'V23S', 'S32V', 'L56D', 'D65L', 'R63B', 'B36R', 'Y13R', 'R31Y', 'J78O', 'O87J', 'C18X', 'X81C', 'X57F', 'F75X', 'K11Q', 'Q11K', 'K32R', 'R23K', 'G21U', 'U12G', 'A92O', 'O29A', 'P94X', 'X49P', 'I70X', 'X07I', 'B68G', 'G86B', 'Q48N', 'N84Q', 'F95D', 'D59F', 'V40D', 'D04V', 'F66E', 'E66F', 'K88F', 'F88K', 'U92P', 'P29U', 'O96T', 'T69O', 'K16O', 'O61K', 'K32I', 'I23K', 'B88L', 'L88B', 'S42U', 'U24S', 'G92L', 'L29G', 'R96W', 'W69R', 'D51V', 'V15D', 'I45Y', 'Y54I', 'O88A', 'A88O', 'P11H', 'H11P', 'Q30R', 'R03Q', 'T39N', 'N93T', 'M98P', 'P89M', 'S16X', 'X61S', 'Z30F', 'F03Z', 'B12G', 'G21B', 'H33Z', 'Z33H', 'S71N', 'N17S', 'X36T', 'T63X', 'K21K', 'K12K', 'G32E', 'E23G', 'R70P', 'P07R', 'V19Q', 'Q91V', 'D39G', 'G93D', 'K43N', 'N34K', 'K78J', 'J87K', 'E82Z', 'Z28E', 'P20V', 'V02P', 'X98S', 'S89X', 'X56N', 'N65X', 'E63M', 'M36E', 'G94Z', 'Z49G', 'C85C', 'C58C', 'I67J', 'J76I', 'N74O', 'O47N', 'O10Z', 'Z01O', 'R64C', 'C46R', 'L84T', 'T48L', 'A80U', 'U08A', 'T88J', 'J88T', 'S77O', 'O77S', 'K56C', 'C65K']

Validation split: ['F32Q', 'Q23F', 'T90M', 'M09T', 'V34K', 'K43V', 'A36O', 'O63A', 'W67S', 'S76W', 'Y66M', 'M66Y', 'I77J', 'J77I', 'A66S', 'S66A', 'R57K', 'K75R', 'B61D', 'D16B', 'V16A', 'A61V', 'T62I', 'I26T', 'O10W', 'W01O', 'I92X', 'X29I', 'Z45Y', 'Y54Z', 'C16G', 'G61C', 'L10E', 'E01L', 'X14A', 'A41X', 'S84F', 'F48S', 'X98Z', 'Z89X', 'W33L', 'L33W', 'E45Q', 'Q54E', 'N66F', 'F66N', 'N68E', 'E86N']

Test split: ['X88U', 'U88X', 'N99Z', 'Z99N', 'J83Z', 'Z38J', 'P69J', 'J96P', 'S84C', 'C48S', 'S57O', 'O75S', 'V11G', 'G11V', 'A31H', 'H13A', 'X38A', 'A83X', 'O38N', 'N83O', 'I58N', 'N85I', 'T22H', 'H22T', 'P63X', 'X36P', 'F10I', 'I01F', 'C33I', 'I33C', 'H50P', 'P05H', 'E39Z', 'Z93E', 'X85I', 'I58X', 'V29V', 'V92V', 'Y78H', 'H87Y', 'E47O', 'O74E', 'A40E', 'E04A', 'V22O', 'O22V']
